# The efficacy of transversus abdominus plane (TAP) blocks in reducing pain scores in dogs undergoing elective spay procedures

**DOI:** 10.18849/ve.v10i1.703

**Published:** 2025-03-18

**Authors:** Francesca Alsworth, Emma Place

**Keywords:** anaesthesia, analgesia, canine, local anaesthesia, neutering, ovariohysterectomy, ovariectomy, spay, tap block

## Abstract

**PICO question:**

In dogs undergoing an elective spay procedure (ovariohysterectomy or ovariectomy), does the use of a transversus abdominus plane (TAP) block compared to non-TAP reduce postoperative pain scores?

**Clinical Bottom Line:**

**Category of research:**

Treatment.

**Number and type of study designs reviewed:**

Three studies were appraised, all of which were prospective, blinded, randomised, controlled trials.

**Strength of evidence:**

Moderate.

**Outcomes reported:**

The available studies deemed a reduction in postoperative pain scores as a marker of treatment efficacy.

**Conclusion:**

There is moderate evidence to suggest that, when compared to a control group, the use of a TAP block reduces postoperative pain scores, in dogs undergoing elective spay procedures (ovariohysterectomy or ovariectomy). The need for postoperative analgesia was reduced when a TAP block had been administered prior to the spay procedure.

## How to apply this evidence in practice

The application of evidence into practice should take into account multiple factors, not limited to: individual clinical expertise, patient’s circumstances and owners’ values, country, location or clinic where you work, the individual case in front of you, the availability of therapies and resources.

Knowledge Summaries are a resource to help reinforce or inform decision making. They do not override the responsibility or judgement of the practitioner to do what is best for the animal in their care.

## Clinical scenario

You are reviewing the anaesthetic and analgesia protocols for elective bitch spay procedures at your clinic, where both ovariectomy and ovariohysterectomy surgeries are performed by either a ventral midline laparotomy or laparoscopic approach. Your colleague suggests administering a transversus abdominis plane (TAP) block prior to spay procedures, and you would like to know if this would reduce postoperative pain scores.

## The evidence

The literature searches returned three prospective, randomised control trials (Espadas-González et al., 2022; Cavaco et al., 2022; Campoy et al., 2022) which were relevant to this PICO. All three studies compared a control group of non-TAP receiving dogs to a TAP receiving group, undergoing a spay procedure. One study then compared two different local analgesic agents within the TAP groups (Campoy et al., 2022). Only one of the studies used a well-defined placebo (Cavaco et al., 2022). Appraisal of these papers showed moderate evidence that the use of either a two or a four point TAP block significantly reduces postoperative pain scores.

## Summary of the evidence

**Campoy et al. (2022):** Transverse abdominis plane injection of bupivacaine with dexmedetomidine or a bupivacaine liposomal suspension yielded lower pain scores and requirement for rescue analgesia in a controlled, randomized trial in dogs undergoing elective ovariohysterectomy **Aim: **To determine the duration and analgesic quality of bupivacaine mixed with dexmedetomidine, or bupivacaine liposome suspension administered as a transverse abdominis plane (TAP) block, compared with a negative control group, in female dogs undergoing elective ovariohysterectomy.

Population:	Female shelter dogs undergoing elective ovariohysterectomy at Cornell University’s Primary Care Surgery Service (USA). Dogs were classified as American Society of Anesthesiologists (ASA) status 1 or 2 based on a physical exam and basic blood work.
Sample size:	26 dogs.
Intervention details:	Dogs were randomly assigned into one of three groups. BUP-DEX: dogs who received a TAP block with bupivacaine and dexmedetomidine block with 0.25% bupivacaine enhanced with dexmedetomidine at 0.5 μg/ml (0.5% bupivacaine with 1 μg/ ml dexmedetomidine was diluted with 0.9% saline [NaCl] solution in a 50:50 dilution) at a total volume of 0.8 ml/kg (n = 9). BLS: dogs who received a TAP block with a bupivacaine liposome injectable suspension containing 13.3 mg/ml bupivacaine at 0.4 ml/kg expanded with 0.5% bupivacaine potentiated with 1 μg/ml dexmedetomidine at 0.4 ml/kg (n = 9). CTRL: dogs who acted as negative controls and received no TAP block or sham treatment (not defined) (n = 8). All dogs received the same general anaesthetic technique for their ovariohysterectomy, which was performed by veterinary students.
Study design:	Randomised control trial. Pain assessors were blinded.
Outcome studied:	Pain scores were evaluated using a Short Form of the Glasgow Composite Measure Pain Scale (GCMPS-SF), at baseline and 4, 6, 12, 24, 48, 72, and 96 hours after time 0 (when the TAP block was applied) by 3 scorers and rescue analgesia instigated if the pain score was greater than 5. Sedation was also evaluated at each time point. Time to return to eating, drinking, urinating, and defaecating. The above variables were compared between the 3 groups.
Main findings (relevant to PICO question):	The proportion of dogs that needed rescue analgesia was significantly higher for the CTRL group (7/9 dogs), than the BUP-DEX (4/9 dogs) and BLS group (3/9 dogs). Time to the need of rescue analgesia was significantly shorter for the CTRL group with all 7 dogs in thegroup needing it by 4 hours after time 0, compared to 3/4 needing it by 4 hours in the BUP-DEX and all 3 dogs in the BLS group. The use of a TAP block results in lower pain scores and therefore a lower need for rescue analgesia postoperatively.
Limitations:	There was no system for scoring visceral pain (no deep palpation). If rescue analgesia was required, it was noted at the next evaluation point, but the study does not describe how it may have affected subsequent pain scores. Surgery was performed by different veterinary students, who were less experienced at the surgical procedure—surgeon variation and experience levels may have influenced pain scores. Although the same scorer assessed each dog throughout the study, overall there were 3 different scorers—which could have resulted in variation between results. The length (i.e. 96 hours post surgery) of pain scoring may not reflect clinical practice, where animals often leave the clinic on the same day postoperatively.

Table note...

**Cavaco et al. (2022):** Analgesic efficacy of ultrasound-guided transversus abdominis plane block in dogs undergoing ovariectomy **Aim: **To determine the analgesic efficacy of bupivacaine administered as a 4-point transverse abdominis plane (TAP) block, compared with a placebo control group, in female dogs undergoing elective ovariectomy.

Population:	Female client-owned dogs undergoing elective ovariectomy. Dogs between 6 months to 3 years were selected, based on an American Society of Anesthesiologists (ASA) status of 1, and a body condition score between 4 and 5. Dogs were excluded if they had any systemic abnormalities or comorbidities. Dogs with behavioural changes, who were pregnant or had received any previous medication were also excluded.
Sample size:	32 dogs.
Intervention details:	Dogs were randomly assigned into two groups: Transversus abdominus plane (TAP) block control (TBC) received a water injection (0.2ml kg^−1^ point) using the 4-point approach (n = 16). TAP block bupivacaine (TBB)- received bupivacaine (0.2 ml kg^−1^ point at 0.25%) using the 4-point approach (n = 16). All dogs received the same premedication and general anaesthetic technique. Surgery was performed 30 minutes after the TAP injection, performed by the same surgeon using the same surgical technique. Postoperatively, if rescue analgesia was required, the patients were re-assessed 30 minutes later and further analgesia given if needed.
Study design:	Randomised control trial. Pain assessors were blinded.
Outcome studied:	Pain scores prior to premedication, and 1, 2, 4, 6, and 8 hours after extubation using the Numerical Rating Scale (NRS) and the short form Glasgow Composite Pain Measurement Scale (GCMPS-SF)**. **Rescue analgesia was given if the score was equal to or above 6 on the GCMPS-SF, or 4 on the NRS. The sedation level was also evaluated. Heart rate, respiratory rate, peripheral oxyhaemoglobin saturation, blood pressure, temperature, end-tidal cardon dioxide and end tidal isoflurane concentration were measured at several points pre, intra, and postoperatively. Serum cortisol levels were also assessed pre, intra, and postoperatively.
Main findings (relevant to PICO question):	There was a significant difference between the groups regarding the need for rescue analgesia within the first 6 hours postoperatively, the need for which was determined by postoperative pain score, with 13/16 dogs in the TBC group needing rescue medication, compared to 1/16 dogs in the TBB group. This study showed that administering bupivacaine in the 4 TAP block points in dogs undergoing ovariectomy promoted adequate postoperative analgesia, significantly reducing postoperative pain scores and therefore the need for systemic rescue analgesia for at least 6 hours postextubation.
Limitations:	A priori power analysis was not utilised. Although the same surgeon carried out the ovariectomies, the authors highlighted that a more experienced surgeon could have reduced pain scores in both groups. It is unclear from the methodology if pain scores were utilised to assess the efficacy of rescue analgesia doses, or how additional rescue analgesia may have impacted subsequent pain scores.

Table note...

**Espadas-González et al. (2022):** Evaluation of the Two-Point Ultrasound-Guided Transversus Abdominis Plane Block for Laparoscopic Canine Ovariectomy **Aim: **To determine the intra and postoperative analgesic efficacy of a 2-point transverse abdominis plane (TAP) block, compared with a negative control group, in female dogs undergoing elective ovariectomy.

Population:	Intact female shelter dogs from Cáceres, Spain, undergoing laparoscopic ovariectomy. Prior to surgery dogs were selected if they were American Society of Anesthesiologists (ASA) category 1 based on physical examination and complete blood work. Dogs were excluded if they were less than 6 months old, pregnant, or showing other comorbidities.
Sample size:	52 dogs.
Intervention details:	26 dogs received general inhalation anaesthesia (control group). 26 dogs received general inhalation anaesthesia as well as a transversus abdominus plane (TAP) block (TAP group), at each injection site, a total volume of 0.3 ml/kg of 0.9% sodium chloride including a dose of 0.5 mg/kg of bupivacaine 0.5% was administered. Dogs were assigned to a group with the use of a random number generator. All dogs underwent laparoscopic ovariectomy using the same technique by the same specialised surgeons, with the same premedication and inhalation anaesthetic protocol.
Study design:	Randomised Control Trial. Pain assessors were blinded.
Outcome studied:	The end tidal isoflurane, heart rate, and mean invasive blood pressure were recorded at 4 points during surgery. The total time to perform the TAP block. Anaesthetic time. Surgical time. Measurement of postoperative pain at 2–3 hours (T1), 6–8 hours (T2) and 20–24 hours (T3) after surgery using the Glasgow Composite Pain Measurement Scale (GCMPS), Melbourne Pain Scale (MPS), and Colorado Pain Scale (CPS), systems and rescue analgesia were administered if the pain score was above 5 on the GCMPS and MPS, or above 3 on the CPS.
Main findings (relevant to PICO question):	This study found that there were significantly lower postoperative GCMPS and MPS pain scores at T1, T2, and T3, and CPS scores at T1 and T2 in the TAP group, compared to the control group. The median GCMPS in the control group at T1 was 1, compared to 0 In the TAP group, 1 in the Control group compared to 0 in the TAP group at T2, and 0 compared to 0 at T3. There was no significant difference in dogs requiring postoperative rescue analgesia—2/26 dogs in the control group, compared to no dogs in the TAP group.
Limitations:	A priori power analysis was not utilised. The study design did not incorporate evaluating visceral pain. If rescue analgesia was required, the study does not mention how this may have impacted subsequent pain scores.The longer anaesthetic time in the TAP group could have biased the results of the pain scores at T1. A rescue dose of propofol was given intra-operatively if the anaesthetist observed a nociceptive response, however propofol does not have analgesic properties.

Table note...

## Appraisal, application and reflection

The transversus abdominis plane (TAP) block has long been utilised in human medicine and aims to infiltrate between the transverse adbominis and the internal oblique muscles, providing analgesia to the ventral and lateral abdominal wall. Several, ideally ultrasound-guided, techniques are reported for surgeries such as caesarean sections and cholecystectomies (Jakobsson et al., 2015).

The TAP block was first described in veterinary medicine in a Canadian Lynx undergoing an exploratory laparotomy for a gastric foreign body (Schroeder et al., 2010) and has since been developed using cadavers. With bitch spays being one of the most common elective surgical procedures undertaken by first opinion veterinarians globally, it is imperative that analgesia is optimised for improved patient welfare, as advocated for by the World Small Animal Veterinary Association (Ryan et al., 2018).

This Knowledge Summary evaluated three studies, all of which were randomised control trials. There are currently no systematic reviews nor meta-analyses relevant to this PICO.

All three studies had well defined test procedures, with similar inclusion criteria. The populations in these studies are reflective of the patients presented for spay procedures in first opinion practice. There was variation in the TAP block technique used. Espadas-González et al. (2022) and Campoy et al. (2022) used a two-point technique, whereas Cavaco et al. (2022) used a 4-point approach.

In all the studies reviewed (Campoy et al., (2022); Cavaco et al., (2022); Espadas-González et al., (2022)), animals scoring less than 5 or 6 on the Glasgow Composite Pain Scale (GCMPS) did not require further analgesia, showing that their pre-operative analgesic protocol (TAP vs non-TAP) was effective in keeping their postoperative pain score below threshold. The efficacy of administering a TAP blocks pre-operatively is reflected by both a reduction in postoperative pain scores, as well as the proportion of those pain scores that reached the threshold and needed actioning with rescue analgesia.

All three studies reported a significant reduction in postoperative pain scores between control and TAP-receiving groups. Both Campoy et al. (2022) and Cavaco et al. (2022) reported a higher need for rescue analgesia in the control groups. In Campoy et al. (2022), 7/9 dogs in the control group required rescue analgesia, compared to a total of 7/18 dogs in the TAP groups. In Cavaco et al. (2022) 13/16 dogs in the control group required rescue analgesia, compared to 1/16 in the TAP group. In these studies, not only were the postoperative pain scores higher in the control group, but they were high enough to require intervention with rescue analgesia, highlighting the benefit of a TAP-block pre-operatively. Espadas-González et al. (2022) reported a significant reduction in postoperative pain scores in TAP receiving dogs, yet there was no significant difference in the need for rescue analgesia between groups, with 2/26 dogs requiring postoperative analgesia in the control group, compared to no dogs in the TAP group. Whilst the pain scores may have been significantly lower in the TAP group, with only 2/26 animals in the control group meeting threshold and requiring postoperative analgesia, the use of the TAP block considering the benefit reported is questionable.

Espadas-González et al. (2022) was the only study that utilised a non-steroidal anti-inflammatory drug as part of its immediate postoperative analgesia protocol. This is not reflective of the majority of bitch spay anaesthetic protocols seen in first-opinion practice, making the results of Cavaco et al. (2022) and Campoy et al. (2022) less applicable to the population in question.

Both Campoy et al. (2022) and Espadas-González et al. (2022) did not use a placebo group, so assessors may have noted a difference between patients as the control group were not mentioned to have been clipped or prepared in the same way as the TAP receiving group. Cavaco et al. (2022) used a placebo group who received water for injection rather than local anaesthetic in their TAP Block.

Whilst all three studies utilised the GCMPS in order to evaluate postoperative pain, Cavaco et al. (2022) also used the Numerical Rating Scale, and Espadas-González et al. (2022) used the Melbourne Pain Scale and Colorado Pain Scale systems. Espadas-González et al. (2022) assessed pain scores up to 24 hours postoperatively, with Campoy et al. (2022) evaluating pain after 96 hours, which is not reflective of general practice, where most patients are discharged on the day of admission.

In conclusion, all three studies found that the use of a TAP block reduced postoperative pain scores for bitch spay procedures; however, it must be noted that there was variation in the techniques used and surgeries performed. Campoy et al. (2022) and Cavaco et al. (2022) found that the need for postoperative rescue analgesia was significantly lower in the TAP block groups, whereas Espadas-González et al. (2022) did not.

It should be recognised that the TAP block is a specialised technique which is currently rarely used in first-opinion small animal veterinary practice, with complications including intraperitoneal injection, puncture of abdominal organs, and liver laceration. All the studies were performed in referral or university centres. The use of TAP blocks in first opinion practice would improve animal welfare, but proper training should be undertaken to reduce complications.

## Methodology

**Search Strategy:** 

	Search strategy
Databases searched and dates covered:	CAB Abstracts (on OVID) 1973 to April 2024 Medline (on OVID) 1946 to April 2024
Search terms:	CAB Abstracts:exp dogs/(dog* or canine* or bitch*).tw.1 or 2ovariectomy/ or ovariectomized females/ or ovariohysterectomy//li&gt;(ovari* or oophorectom* or spay* or hysterectom*).tw.4 or 5exp amides/(nerve block* or bupivacain* or transversus abdominis plane* or TAP or chemical neurolyses or chemodenervation* or regional analgesi* or amide* or marcain* or sen- sorcain*).tw.7 or 83 and 6 and 9limit 10 to english language Ovid MEDLINEDogs/(dog* or canine* or bitch*).tw.1 or 2hysterectomy/ or exp ovariectomy/(ovari* or oophorectom* or spay* or hysterectom*).tw.4 or 5Nerve Block/exp amides/ or exp anilides/ or exp bupivacaine/9 (nerve block* or bupivacain* or transversus abdominis plane* or TAP or chemical neurolyses or chemodenervation* or regional analgesi* or amide* or marcain* or sen- sorcain*).tw.7 or 8 or 93 and 6 and 10limit 11 to english language
Dates searches performed:	08 April 2024

**Exclusion / Inclusion Criteria:** Caption placeholder

Exclusion:	Papers irrelevant to the PICO question. Papers discussing different locoregional anaesthetic or analgesic techniques for spay procedures. Articles not written in the English language.
Inclusion:	Studies that compared a TAP group to a non-TAP group for dogs undergoing a spay procedure. Randomised control trials.

**Search Outcome:** 

Database	Number of results	Excluded – different anaesthetic or analgesic technique	Excluded – not answering the PICO	Total relevant papers
CAB Abstracts	84	25	56	3
Ovid MEDLINE	111	35	73	3
Total relevant papers when duplicates removed				3

## ORCID

Francesca Alsworth: https://orcid.org/0009-0007-3430-3439

Emma Place: https://orcid.org/0000-0001-8326-3604

## Conflict of Interest

The authors declare no conflicts of interest.

## Disclaimer

Knowledge Summaries are a peer-reviewed article type which aims to answer a clinical question based on the best available current evidence. It does not override the responsibility of the practitioner. Informed decisions should be made by considering such factors as individual clinical expertise and judgement along with patient’s circumstances and owners’ values. Knowledge Summaries are a resource to help inform and any opinions expressed within the Knowledge Summaries are the author's own and do not necessarily reflect the view of the RCVS Knowledge. Authors are responsible for the accuracy of the content. While the Editor and Publisher believe that all content herein are in accord with current recommendations and practice at the time of publication, they accept no legal responsibility for any errors or omissions, and make no warranty, express or implied, with respect to material contained within. For further information please refer to our Terms of Use.
